# Verrucous carcinoma of the head and neck – not a human papillomavirus-related tumour?

**DOI:** 10.1111/jcmm.12211

**Published:** 2013-12-18

**Authors:** Katarina Odar, Boštjan J Kocjan, Lea Hošnjak, Nina Gale, Mario Poljak, Nina Zidar

**Affiliations:** a Faculty of Medicine, Institute of Pathology, University of LjubljanaLjubljana, Slovenia; b Faculty of Medicine, Institute of Microbiology and Immunology, University of LjubljanaLjubljana, Slovenia

**Keywords:** verrucous carcinoma, head and neck, human papillomaviruses, polymerase chain reaction, p16 protein

## Abstract

Association between verrucous carcinoma (VC) of the head and neck and human papillomaviruses (HPV) is highly controversial. Previous prevalence studies focused mostly on α-PV, while little is known about other PV genera. Our aim was to investigate the prevalence of a broad spectrum of HPV in VC of the head and neck using sensitive and specific molecular assays. Formalin-fixed, paraffin-embedded samples of 30 VC and 30 location-matched normal tissue samples were analysed, by using six different polymerase chain reaction-based methods targeting DNA of at least 87 HPV types from α-PV, β-PV, γ-PV and μ-PV genera, and immunohistochemistry against p16 protein. α-PV, γ-PV and μ-PV were not detected. β-PV DNA was detected in 5/30 VC (16.7%) and in 18/30 normal tissue samples (60.0%): HPV-19, -24 and -36 were identified in VC, and HPV-5, -9, -12, -23, -24, -38, -47, -49 and -96 in normal tissue, whereas HPV type was not determined in 2/5 cases of VC and in 6/18 normal tissue samples. p16 expression was detected in a subset of samples and was higher in VC than in normal tissue. However, the reaction was predominantly cytoplasmic and only occasionally nuclear, and the extent of staining did not exceed 75%. Our results indicate that α-PV, γ-PV and μ-PV are not associated with aetiopathogenesis of VC of the head and neck. β-PV DNA in a subset of VC and normal tissue might reflect incidental colonization, but its potential biological significance needs further investigation.

## Introduction

Human papillomaviruses (HPV) are a heterogenous group of epitheliotropic DNA viruses from the *Papillomaviridae* family, consisting of more than 170 different types. They cluster into five genera: *Alphapapillomavirus* (α-PV), *Betapapillomavirus* (β-PV), *Gammapapillomavirus* (γ-PV), *Mupapillomavirus* (μ-PV) and *Nupapillomavirus* (ν-PV). Human papillomaviruses can be found as commensals colonizing human epithelia, but their clinical importance lies in association with several cutaneous and mucosal lesions varying from benign warts to several types of cancer. According to preferential site of infection, HPV have been traditionally divided into »mucosal« types (α-PV), predominantly found in anogenital lesions and »cutaneous« types (β-PV, γ-PV, μ-PV, ν-PV and a subset of α-PV types), typically isolated from skin and hair follicles. The clinically most important genus α-PV associates more than 40 different HPV types that can be further divided into low- and high-risk types defined by their risk of progression to malignancy. Low-risk types (the most important being HPV-6 and HPV-11) are mainly associated with benign lesions, such as anogenital warts and laryngeal papillomas, and high-risk types (the most important being HPV-16) play a leading role in the development of cervical, anal and vaginal carcinoma, and a proportion of other cancers, including a subset of squamous cell carcinoma (SCC) of the head and neck 1–3.

Verrucous carcinoma (VC) is a rare variant of well-differentiated SCC. It arises most commonly on the mucosa of the head and neck, predominantly in the oral cavity and the larynx, but it can also arise on other locations, such as the skin, anogenital region, urinary bladder and oesophagus. Verrucous carcinoma is characterized by an exophytic, wart-like macroscopic appearance. Microscopically, it consists of filiform projections, lined by thick, well-differentiated keratinizing squamous epithelium, composed of one to a few layers of basal cells and multiplied, voluminous cells of spinous type, lacking cytologic atypia. The invasive nature of VC is difficult to assess, but at the stromal interface, it always exhibits a well-defined, pushing margin. It grows slowly and even though it is locally destructive, it is believed to be unable to metastasize. The prognosis is therefore significantly better in VC than in conventional SCC 4.

The aetiopathogenesis of VC of the head and neck has not been fully elucidated. It is generally believed that conventional SCC and VC share similar aetiological factors 4, but certain studies indicate that their molecular background differs 5,6. The involvement of HPV in the pathogenesis of VC is controversial. Even though this association has been commonly quoted in the literature 7,8, the reported prevalence of HPV in VC ranges from 0% to 100% 7,9–31 (Table 1). The majority of previous studies have focused on α-PV in VC 7,10–25,27–30, but little is known about the prevalence of HPV types from other genera.

**Table 1:** Overview of studies investigating human papillomaviruses in verrucous carcinoma of the head and neck. To summarize, a total of 336 cases were included, and HPV was detected in 119 cases (35.4%). Single case reports are not included

Reference	Sample location/number (sample type)	Method of HPV detection & typing [identifiable HPV types]	Detected HPV types (overall HPV prevalence in%)		Summary of relevant findings, comments and conclusions
			VC	NC	
Abramson *et al*. 10	Larynx/5 VC, 3 NC (frozen)	IHC [1, broadly cross reactive], DH [11, 16, 18]	IHC neg, 16 by DH (100%)	IHC neg, 16 by DH (100%)	Laryngeal VC are HPV-associated. HPV may cause anaplastic transformation after radiotherapy
Adler-Storthz *et al*. 11	Oral cavity/16 VC (FFPE)	ISH [2, 6, 16]	2 (12.5%)	NA	HPV-2 may play a role in pathogenesis of oral VC, but may be an incidental finding
Greer *et al*. 12	Oral cavity/20 VC (FFPE)	ISH [6, 11, 16, 18, 31, 33, 35]	6, 16 (20%)	NA	Higher prevalence of HPV in VC than in SCC and other lesions. HPV of known type can be detected in oral malignant and premalignant lesions
Pérez-Ayala *et al*. 13	Larynx/3 VC, 6 NC	PCR (specific primers) & DH [6, 11]	16 (100%)	16 (50%)	High prevalence of HPV in VC. HPV-16 may act as a cofactor in laryngeal carcinogenesis in general
Johnson *et al*. 14	Head and neck/11 VC (FFPE)	ISH [HPV DNA], PCR (specific primers) & DH [6, 11, 16, 18]	neg	NA	No evidence of HPV in VC of the head and neck
Young and Min 15	Oral cavity/10 VC (FFPE)	ISH [6, 11, 16, 18, 31, 33, 35]	neg	NA	Inconsistency in demonstration of HPV in oral cancer. No specific comment on VC
Holladay and Gerald 16	Oral cavity/2 VC, 15 NC (FFPE)	PCR (MY09/11) & DH [6, 11, 16, 18, 33]	neg	16 (33.3%)	Little difference in HPV prevalence between various nonmalignant, premalignant and malignant lesions. HPV alone not sufficient for oral carcinogenesis. No comment on VC
Kasperbauer *et al*. 17	Larynx/20 VC (FFPE)	PCR (MY09/11) & no typing, ISH [6, 11, 16, 18, 31, 33, 35]	HPV DNA by PCR (85%), neg by ISH	NA	Result supports the role of HPV in laryngeal VC. ISH is inadequate to detect HPV in VC
Noble-Topham *et al*. 18	Oral cavity/29 VC (FFPE)	PCR (specific primers) & DH [6b/11, 16, 18]	6b/11,16, 18 (41%)	NA	HPV-18 may play a role in tumorigenesis of oral VC. PCR is effective for detection of HPV in VC
Shroyer *et al*. 19	Oral cavity/17 VC (FFPE)	PCR (MY09/11) & DH (broad spectrum & specific probes) [6, 11, 16, 18, 33], ISH [6/11, 16/18, 31/33/35]	6/11 by PCR (41%) 6/11 by ISH (41%)	NA	High proportion of oral VC associated with HPV-6 and -11
Anderson *et al*. 20	Oral cavity/8 VC (FFPE, frozen)	PCR (MY09/11) & DH [16/18]	16/18 (25%)	NA	HPV may participate in pathogenesis of oral VC
Fliss *et al*. 21	Larynx/29 VC, 4 NC (FFPE)	PCR [6, 11, 16, 18], SB [16,18]	16, 18 (45%)	16 (25%)	HPV may be important in pathogenesis of some laryngeal VC
Multhaupt *et al*. 22	Larynx/11 VC (FFPE)	ISH [6, 11, 16, 18, 31, 33, 35, 42-45, 51, 52]	neg	NA	Laryngeal VC is not related to HPV
Balaram *et al*. 23	Oral cavity/15 VC (frozen, FFPE)	PCR (consensus and specific primers) & seq [6, 11, 16, 18]	6, 11, 16, 18 (67%)	NA	High prevalence of HPV in oral cancer in India. Lower prevalence in VC than in SCC
López-Amado *et al*. 24	Larynx/10 VC (FFPE)	IHC & no typing [HPV antigen]	NS (40%)	NA	HPV is well recognized as aetiological factor for VC. Present study found HPV in a high percentage of VC
Orvidas *et al*. 25	Nasal cavity/7 VC (FFPE)	PCR (MY09/11, GP5+/6+) & seq	neg	NA	Role of HPV in aetiology of nasal VC not confirmed
Mitsuishi *et al*. 26	Lip/5 VC (frozen)	Various PCR-based methods for detection & typing [mucosal and cutaneous HPV]	X, 2, 20, 27, 57, 62 (100%)	NA	Various mucosal and cutaneous HPV associated with pathogenesis of VC of the lip
Szentimary *et al*. 7	Head and neck/12 VC (NS)	NS [α-PV DNA]	α-PV (100%)	NS	Association between HPV and head and neck carcinomas with basaloid and verrucous features
Gonzalez *et al*. 27	Oral cavity/9 VC (FFPE), 60 NC (swabs)	PCR (MY09/11) & RFA [44 types, mostly α-PV] or PCR (GP5+/6+) & DH [6, 11, 16, 18, 31, 33, 35]	6, 11, 16 (88.9%)	neg	Results support aetiological role of HPV in at least a subset of oral cancer
Fujita *et al*. 28	Oral cavity/23 VC, 10 NC (FFPE)	PCR (SPF) & seq [at least 43 types], ISH [6, 11, 16, 18, 31, 33]	18, 6, 74, 11, 33 (48%) by PCR, NS (26%) by ISH	6, 11, 18 (70%) by PCR, ISH neg	Multiple HPV infections may participate in histogenesis of oral VC. No significant difference in HPV prevalence between VC and non-neoplastic lesions. HPV in non-neoplastic lesions are inactive
Lin *et al*. 29	Oral cavity/48 VC (FFPE)	IHC [16/18 E6 protein]	neg (low labelling indices)	NA	No participation of HPV 16/18 in oral VC
Saghravanian *et al*. 30	Oral cavity/21 VC, 18 NC (FFPE)	PCR (GP5+/6+) & specific PCR [16, 18, 31, 33]	16, 18 (14.3%)	neg	No significant relationship between VC and HPV
del Pino *et al*. 31	Head and neck/5 VC (FFPE)	Various PCR-based methods for detection & typing [83 types-α-PV, β-PV, γ-PV, μ-PV, ν-PV	35, 45 (20%)	NA	Results do not support causal role of HPV in VC

DH: DNA hybridization (dot/Southern/slot blot); FFPE: formalin fixed, paraffin embedded; GP1/2, GP5**+**/6**+**, MY09/11, SPF: primers targeting a spectrum of mostly α-PV types; IHC: immunohistochemistry; ISH: *in situ* hybridization; NA: not analysed; NC: non-neoplastic control tissue; neg: negative; NS: not specified; PCR: polymerase chain reaction; RFA: restriction fragment analysis; SCC: squamous cell carcinoma; seq: DNA sequencing; VC: verrucous carcinoma; X: undetermined HPV type.

To elucidate the controversial aetiologic role of HPV in VC of the head and neck, and to investigate the prevalence of HPV genera other than α-PV in this tumour, we performed a comparative study of the HPV prevalence in VC and normal tissue of the head and neck, using six different PCR protocols for detection of at least 87 HPV types from α-PV, β-PV, γ-PV and μ-PV genera. In addition, an immunohistochemical analysis of p16 protein expression was performed.

## Materials and methods

The study was approved by Ethical Commission of Republic of Slovenia.

### Patients and tissue samples

Surgical biopsy samples of cases of VC of the head and neck were included, diagnosed at the Institute of Pathology, Faculty of Medicine, University of Ljubljana during a period of 10 years. In all of them, routine diagnosis of VC was made on the basis of the following criteria 4: the presence of club-shaped papillary projections and invaginations of well-differentiated squamous epithelium with enlarged spinous cells and marked surface keratinization, with no atypia and with rare mitoses, observed only in the basal and parabasal cells.

For the purpose of this study, all biopsies were reviewed by two pathologists (N.G. and N.Z.). Those containing foci of ‘conventional’ SCC were not included in the study, as well as cases in which not enough tissue was available for immunohistochemistry and molecular analyses.

As normal tissue controls, biopsy samples of microscopically normal mucosa and skin were included, obtained from patients who underwent surgery for various conditions other than VC. The normal tissue samples were matched to VC by location.

All tissue samples were routinely collected during surgery, fixed in 10% buffered formalin for 24 hrs and embedded in paraffin. Haematoxylin and eosin stains were made for routine histopathological diagnostics. For the purposes of our study, representative tissue blocks were retrieved from archives of the Institute of Pathology, Faculty of Medicine, University of Ljubljana.

### Human papillomavirus DNA detection and typing

Total DNA was extracted from the tissue blocks sectioned at 10 μm, as previously described 32,33. When necessary, manual dissection was performed prior sectioning, to assure that only relevant parts of the tissue blocks were submitted for further analyses 5,6. To control the potential contamination during sectioning, several protective measures were used 32. The microtome blade and surface were cleaned after each sample using xylene and DNA AWAY surface decontaminant (Thermo Fisher Scientific, Waltham, MA, USA), and HPV-negative control sample (normal human liver) was sectioned alternately at the beginning of the series and after every three tissue blocks. The extracted DNA was quantified on a NanoDrop ND-2000c spectrophotometer (NanodDrop Technologies, Oxfordshire, UK) and stored at −20°C until use.

Several »in-house« and commercially available PCR-based methods were used to detect DNA of at least 87 HPV types from the genera α-PV, β-PV, γ-PV and μ-PV. For all PCR experiments, 50–100 ng (5 μl) of DNA was used per a 25 μl reaction 34. To control the potential amplicon carryover contamination, water blanks were placed after every seventh reaction tube in all PCR runs. The quality of each DNA sample was verified by real-time PCR amplification of a 268-bp fragment of human beta-globin gene 35. The amplification products of all »in-house« PCRs were analysed by electrophoresis on PCR CheckIT Wide Mini S-2x25 gels (Elchrom Scientific, Zurich, Switzerland). Human papillomavirus types were determined by direct sequencing of PCR products 36, unless indicated otherwise.

For the detection of α-PV, a touchdown GP5+/GP6+ PCR protocol 34 with an additional HPV-68-specific primer 37 was used, targeting a 150-bp fragment of L1 gene of at least 41 different HPV types, including HPV-6, -11, -13, -26, -16, -18, -30, -31, -32, -33, -34, -35, -39, -40, -42, -43, -44, -45, -51, -52, -53, -54, -55, -56, -57, -58, -59, -61, -66, -68, -70, -71, -72, -73, -81, -82, -83, -84, -89, -90 and -91. To specifically detect »low-risk« α-PV associated with different mucosal and cutaneous warts, a novel set of primers was developed (Table 2), targeting an approximately 190-bp fragment of E1 gene of HPV-2, -3, -6, -7,-10, -13, -11, -27, -28, -29, -32, -40, -42, -43, -44, -57, -74, -77, -78, -91, -94, -117 and -125. The PCR protocol was set up on a GeneAmp PCR System 9700 (PE Applied Biosystems, Foster City, CA, USA) and performed with a HotStarTaq *Plus* DNA Polymerase kit (Qiagen, Hilden, Germany). The reaction mixture contained template DNA, 2.5 μl of 10× CoralLoad PCR Buffer with 15 mM MgCl_2_, 200 μM of dNTPs, 0.625 U of HotStarTaq *Plus* DNA polymerase, 0.5 μM of each of the primers and water up to 25 μl. The cycling conditions were 95°C for 5 min., followed by 40 cycles of 94°C for 30 sec., 50°C for 1 min. and 72°C for 1 min., followed by final extension at 72°C for 10 min. and cooling the reaction mixture to 4°C. To definitely exclude the presence of clinically most important α-PV types, all the samples were additionally tested with a reverse line-blot hybridization-based INNO-LIPA HPV Genotyping Extra *Amp* test (Innogenetics, Gent, Belgium), capable of recognizing 28 different HPV types (HPV-6, -11, -16, -18, -26, -31, -33, -35, -39, -40, -43, -44, -45, -51, -52, -53, -54, -56, -58, -59, -66, -68, -69/71, -70, -73, -74 and -82). The test was performed strictly following the manufacturer's instructions.

**Table 2:** List of primers for detection of »low-risk« human papillomaviruses from the genus Alpha

Primer	Nucleotide sequence (5′-3′)	Nucleotide positions*
6-11-44F(50,8)	GCAGGACCTAAAACGAAA	1068-1085
2-27-57F(51,55)	CAGGCCCTAAAACGAAA	1069-1085
42F(51,13)	TACAGGCACTAAAACGAAAG	1067-1086
3-125F(51,49)	TGCAGACTGTAAAACGAAAG	1067-1086
28-77F(51,8)	TTCAGGCTGTAAAACGAAA	1067-1085
7-40F(51,15)	GTGCGAGTTAAAGACGAAAG	1068-1086
43F(51,8)	GCAGGAGTTAAAACGAAAGT	1068-1086
6_11_42R(50,95)	TTCCACTTCAGAATAGCCA	1260-1242
44_43R(51,63)	TTCCACTTCAGTATTGCCA	1260-1242
2-27-57R(51,28)	ACCATCTGCGTAGTTGCC	1259-1243
3-125R(50,45)	ATCCACCTGTGTTTGGC	1260-1244
7R(51,63)	TTCCACTTGTGAATAGCCA	1260-1242

* Relative to the reference HPV-6 genome sequence (X00203).

For the detection of β-PV, a reverse line-blot hybridization-based RHA Kit Skin (beta) HPV test (Diassay BV, Rijswijk, The Netherlands) was used, enabling detection of at least 25 different HPV types, including HPV-5, -8, -9, -12, -14, -15, -17, -19, -20, -21, -22, -23, -24, -25, -36, -37, -38, -47, -49, -75, -76, -80, -92, -93 and -96. The assay was performed as instructed by the manufacturer, except that the number of PCR cycles was increased from 35 to 40.

For the detection of γ-PV, a C4F/C4R PCR protocol was used 38, targeting a 330-bp fragment of L1 gene of at least five different HPV types: HPV-4, -48, -50, -60 and -65.

For the detection of HPVs from the genus μ-PV (HPV-1 and -63), a SK-PCR was used 39, targeting 210–238-bp fragments of L1 gene of a wide spectrum of HPV associated with different cutaneous warts (HPV-1, -2, -3, -4, -7, -10, -27, -28, -29, -40, -57, -60, -63, -65, -77, -91 and -94).

### Immunohistochemistry

Immunohistochemistry was performed on all samples. Tissue sections were cut from the paraffin blocks at 4–5 μm. Staining was performed in the BenchMark XT immunostainer (Ventana Medical Systems, Tuscon, AZ, USA). After antigen retrieval with Cell Conditioning 1 (CC1) buffer (Ventana Medical Systems) at 95°C for 30 min., the slides were incubated with primary antibodies against p16 (clone E6H6, CINtec Histology, mtm Laboratories, Heidelberg, Germany) for 30 min. Immunoreactivity was visualized using the iVIEW DAB Detection Kit (Ventana Medical Systems), according to the manufacturer's instructions. Sections were counterstained with haematoxylin.

The reaction was scored as follows: 0 (<5% of positive cells), 1 (>5% and <25% of positive cells), 2 (>25% and <50% of positive cells), 3 (>50% and <75% of positive cells), 4 (>75% of positive cells). The pattern of staining was also analysed (cytoplasmic, nuclear). Cells with nuclear or cytoplasmic staining or both were considered positive.

### Statistical analyses

For statistical analyses, Fisher's exact test was used. Differences were considered statistically significant when *P* < 0.05.

## Results

Thirty cases of VC were included. There were 21 males and nine females, aged 31–83 years (mean 60.63). The tumours were located in the oral cavity (15 cases), in the larynx (10 cases), in the nasal cavity (two cases) and on the skin (three cases). In the control group, there were 30 samples of the normal mucosa and skin, matched to VC by location, samples were obtained from 13 males and 17 females, aged 19–83 years (mean 53.47).

### Prevalence of human papillomavirus DNA in verrucous carcinoma and in the normal mucosa and skin of the head and neck

The 268-bp fragment of human beta-globin gene was successfully amplified in all tissue samples included in the study. Human papillomaviruses DNA was not detected in any of the HPV-negative control samples (normal human liver). All water blanks used to control for PCR contaminations were negative for internal control amplification.

Results of the HPV detection and typing are summarized in Tables 3 and 4. α-PV, γ-PV and μ-PV DNA was not detected in any of the samples. Using RHA Kit Skin (beta) HPV test (Diassay BV), β-PV DNA was detected in 5/30 (16.7%) cases of VC and in 18/30 (60.0%) samples of normal mucosa and skin. The difference in β-PV DNA prevalence between VC and normal tissue was statistically significant (*P* = 0.001). Among β-PV positive cases of VC, there were 1/15 case from the oral cavity, 2/10 cases from the larynx and 2/2 cases from the nasal cavity. No β-PV DNA was detected in VC of the skin. Human papillomaviruses-19, -24 and -36 were detected each in one case. In two cases of VC, HPV type was not further determined. Among β-PV positive cases of normal mucosa and skin, there were 11/15 cases from the oral cavity, 4/10 cases from the larynx and 3/3 cases from the skin. HPV-38 and -96 were detected each in two cases and HPV-5, -12, -23, -24, -47 and -49 each in one case. In two cases, two different HPV types were co-present (HPV-9 and -24 in one case, and HPV-38 and -49 in another). In six HPV-positive normal tissue samples, HPV type was not further determined.

**Table 3:** Human papillomaviruses and immunohistochemical expression of p16 in verrucous carcinoma of the head and neck

Localization	Gender, age of patient	HPV status (genus/type)	p16 expression score
Oral cavity	M, 80	β-PV/HPV-19	0
Oral cavity	M, 81	Negative	0
Oral cavity	M, 54	Negative	0
Oral cavity	F, 79	Negative	0
Oral cavity	F, 83	Negative	2
Oral cavity	M, 75	Negative	0
Oral cavity	M, 56	Negative	1
Oral cavity	F, 49	Negative	0
Oral cavity	F, 49	Negative	0
Oral cavity	F, 54	Negative	0
Oral cavity	M, 49	Negative	1
Oral cavity	M, 61	Negative	0
Oral cavity	M, 40	Negative	0
Oral cavity	M, 76	Negative	0
Oral cavity	F, 62	Negative	1
Larynx	M, 53	β-PV/HPV-36	0
Larynx	M, 73	β-PV/HPV-24	0
Larynx	M, 31	Negative	3
Larynx	F, 57	Negative	3
Larynx	M, 46	Negative	2
Larynx	M, 66	Negative	0
Larynx	M, 66	Negative	0
Larynx	M, 56	Negative	3
Larynx	M, 58	Negative	0
Larynx	M, 58	Negative	0
Nasal cavity	F, 69	β-PV/HPV-X	0
Nasal cavity	M, 43	β-PV/HPV-X	1
Skin	M, 70	Negative	1
Skin	M, 64	Negative	1
Skin	F, 61	Negative	1

F: female; HPV: human papillomavirus; HPV-X: genotype could not be further determined; M: male; 0: score 0 (<5% of positive cells); 1: score 1 (>5% and <25% of positive cells); 2: score 2 (>25% and <50% of positive cells); 3: score 3 (>50% and <75% of positive cells).

**Table 4:** Human papillomaviruses and immunohistochemical expression of p16 in normal mucosa and skin of the head and neck

Localization	Gender, age of patient	HPV status (genus/type)	p16 expression score
Oral cavity	F, 22	β-PV/HPV-12	0
Oral cavity	M, 78	β-PV/HPV-5	0
Oral cavity	F, 71	β-PV/HPV-47	0
Oral cavity	F, 52	β-PV/HPV-96	0
Oral cavity	M, 63	β-PV/HPV-X	0
Oral cavity	F, 56	β-PV/HPV-49	0
Oral cavity	F, 24	β-PV/HPV-9, -24	0
Oral cavity	F, 51	β-PV/HPV-X	0
Oral cavity	M, 60	β-PV/HPV-X	0
Oral cavity	F, 60	β-PV/HPV-38	0
Oral cavity	F, 68	β-PV/HPV-X	0
Oral cavity	F, 34	Negative	0
Oral cavity	M, 19	Negative	0
Oral cavity	M, 47	Negative	0
Oral cavity	M, 64	Negative	1
Larynx	F, 71	β-PV/HPV-38	0
Larynx	M, 60	β-PV/HPV-24	0
Larynx	F, 63	β-PV/HPV-96	0
Larynx	M, 57	β-PV/HPV-X	0
Larynx	M, 57	Negative	0
Larynx	F, 34	Negative	0
Larynx	M, 50	Negative	0
Larynx	F, 62	Negative	1
Larynx	M, 53	Negative	0
Larynx	M, 40	Negative	0
Nasal cavity	F, 62	Negative	0
Nasal cavity	M, 41	Negative	0
Skin	F, 69	β-PV/HPV-X	0
Skin	F, 33	β-PV/HPV-23	0
Skin	F, 83	β-PV/HPV-38, -49	0

F: female; HPV: human papillomavirus; HPV-X: genotype could not be further determined; M: male; 0: score 0 (<5% of positive cells); 1: score 1 (>5% and <25% of positive cells).

### Expression of p16 protein in verrucous carcinoma and in the normal mucosa and skin of the head and neck

**Figure 1:**
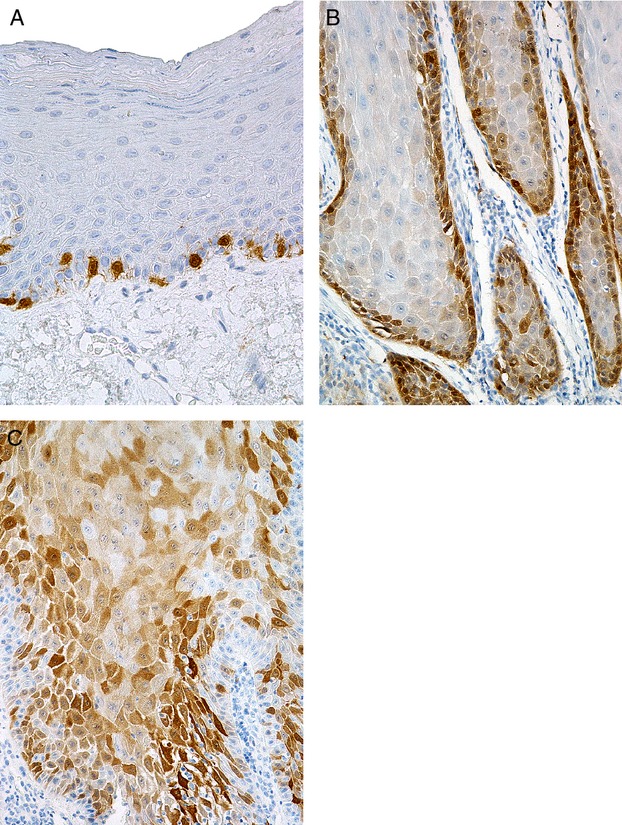
Immunohistochemical expression of p16. (A) Normal epithelium: staining in occasional basal cells. (B) Verrucous carcinoma: staining in basal/parabasal cells. (C) Verrucous carcinoma: more extensive, mosaic staining in basal, parabasal and spinous cells.

Results of immunohistochemical analysis of p16 protein expression are presented in Tables 3 and 4, and in Figure 1. Score 0 (<5% of positive cells) was assigned in 18/30 cases of VC (60%). Among them, only 11 tumours were completely devoid of staining, while single cells with weak to moderate, predominantly cytoplasmic reaction were found in other cases. Score 1 (>5% and <25% of positive cells) was assigned in seven cases (23.3%), score 2 (>25% and <50% of positive cells) in two cases (6.7%), and score 3 (>50% and <75% of positive cells) was assigned in three cases (10%). The reaction in these cases was predominantly cytoplasmic and partly also nuclear. The staining pattern was patchy, mosaic in some cases, and more continuous in others, with intensity of staining varying from weak and moderate to strong in areas with accentuated nuclei. The strongest reaction was found in the basal and parabasal cells. Score 4 (>75% of positive cells) was not assigned in any of the investigated tumours.

In the normal mucosa and skin, score 0 was assigned in all but two cases (6.7%) which were assigned score 1. The reaction was predominantly cytoplasmic, with occasionally accentuated nuclei. The staining pattern was mostly patchy, mosaic, predominantly in the basal/parabasal cells. Scores 2, 3 and 4 were not assigned in any of the normal tissue samples.

The expression of p16 was significantly higher in VC than in normal tissue (*P* = 0.005). However, no statistical difference existed for the expression levels of p16 with scores 3 and above (3/30 *versus* 0/30, *P* = 0.24).

## Discussion

We analysed the prevalence of at least 87 HPV types in VC and normal tissue of the head and neck using six PCR-based assays. α-PV, γ-PV and μ-PV were not detected in any of the samples. β-PV DNA was found in 16.7% of VC and in 60% of normal tissue samples. We also analysed the immunohistochemical expression of p16 protein and found that it was significantly higher in VC than in the normal tissue. However, the reaction was predominantly cytoplasmic and only occasionally nuclear, and the extent of staining never exceeded 75% of cells. These results suggest that VC of the head and neck is not induced by α-PV, γ-PV or μ-PV, while the significance of β-PV DNA in VC remains controversial.

Verrucous carcinoma was named after its characteristic wart-like appearance, leading to the assumption that it is aetiopathogenetically related to HPV infection 19. Since 1980s, several studies have investigated the presence of HPV in VC from various locations of the head and neck (Table 1). Methods of variable sensitivity and specificity were used, and with few exceptions 29, the number of cases was low. Most of these studies focused predominantly on detection and/or typing of only a subset of high- and low-risk »mucosal« α-PV types. Results of these studies are highly controversial. On one hand, several studies detected certain α-PV types in up to 100% of cases, claiming that at least a subset of VC of the head and neck is induced by α-PV 7,10–13,17–21,24,27,28. In contrast, other investigators found no association between VC of the head and neck and HPV infection 14–16,22,25,29,30. Interpretation of these controversial results is challenging, as the differences in reported HPV prevalences can be attributed to various factors such as the type, sensitivity, specificity and performance quality of the HPV detection methods, sample characteristics (formalin-fixed or frozen surgical samples, swab samples), case selection bias, geographical diversity, publication bias (the tendency to promote and publish positive results rather than negative), *etc*. 9,35. Even though some of these factors cannot be completely recognized and eliminated, the fact that α-PV cannot be reproducibly detected in VC of the head and neck is by itself a strong indication that α-PV are not involved in aetiopathogenesis of VC. Our study, performed on a relatively large number of VC, using highly sensitive and specific PCR-based assays, further supports this hypothesis.

Our results of p16 immunohistochemistry also speak against the causal role of α-PV in the pathogenesis of VC. p16 immunohistochemistry is commonly used as a surrogate marker of high-risk α-PV infection in different malignant tumours, including anal, cervical and head and neck SCC. However, as noted in previous studies, it has to be interpreted with caution 40,41. Only SCC of the head and neck with greater than 75% of both nuclear and cytoplasmic p16 staining has been consistently identified by other methods as truly HPV-associated 40. The pattern of p16 staining in VC observed in our study does not fit these criteria. Namely, in the p16-positive cases of VC, the staining never exceeded 75% of cells, and the reaction was mostly cytoplasmic and only partially nuclear. Previous studies have already reported a higher extent of p16 staining in VC compared with the normal epithelium 42. The authors speculated that it was a sign of HPV infection in VC, but we demonstrated that this is not the fact. Factors other than α-PV infection have to be associated with the p16 staining patterns in VC observed by previous studies 42 and our own, but these factors are currently unknown.

In addition to the broad spectrum of α-PV, we also analysed the prevalence of β-PV, γ-PV and μ-PV in VC and normal tissue. Interestingly, β-PV DNA was detected in 16.7% of VC and in 60% of the normal tissue samples. This finding is in accordance with some previous studies reporting a relatively high prevalence of β-PV in the skin and oral mucosa in general populations 43,44. It further supports suggestions that the stratification of HPV into »mucosal« and »cutaneous« types is an oversimplification, and that many of the HPV types actually share dual epithelial tropism 44. In addition, our study showed that β-PV can be found not only in the oral cavity 44 but also in other sites of the head and neck mucosa, such as the larynx and nasal cavity. Our finding of β-PV DNA in a subset of VC in addition to a substantially higher prevalence in the normal tissue samples has two potential explanations. It could merely represent an incidental tissue colonization and not a clinically important infection. On the other hand, recent discoveries on SCC of the skin indicate that the low detection rate of β-PV in cancer in contrast to its high prevalence in the normal tissue could be biologically significant. In the normal appearing epidermis, β-PV may act together with other susceptibility factors to promote early stages of carcinogenesis, and are readily detectable in the tissue. However, β-PV genome cannot be maintained in fully developed cancer, and it therefore becomes undetectable with traditional detection methods 2. Additional studies are necessary to further elucidate the potential role of β-PV in the development VC of the head and neck, especially those from mucosal sites.

In contrast to β-PV, we detected no γ-PV and μ-PV DNA in our samples, confirming previous observations that γ-PV and μ-PV are less frequent in the head and neck than β-PV 44. It probably also indicates that VC of the head and neck is not induced by these HPV types. However, as many γ-PV that were previously detected in the oral cavity represented novel HPV types 44, it is also possible that our method was suboptimal for their detection.

Two previous studies investigated a broad spectrum of HPV in VC. Del Pino *et al*. 31 analysed the prevalence of 83 HPV types from the α-PV, β-PV, γ-PV, μ-PV and ν-PV genera in 13 cases of VC, among them five cases from the head and neck mucosa and three cases from the skin. They only found HPV-35/HPV-45 DNA in one VC from the oral cavity, probably unrelated to active infection and concluded that the causal role of HPV in the development of VC is unlikely. In contrast to our study, they did not detect β-PV DNA in any of the VC cases, and their study included no normal tissue controls. Mitsuishi *et al*. 26 investigated a broad spectrum of »mucosal« and »cutaneous« HPV in five cases of VC of the lip. They found α-PV and β-PV DNA in all of the investigated samples and concluded that these viruses play a role in the development of VC of the lip. However, the number of samples in their study was low, and no normal tissue controls were included.

In addition to 30 cases of VC, our study also included 30 normal tissue control samples, matched to VC by location and sample type. With some exceptions 10,13,16,21,28,30, most of the previous HPV prevalence studies on VC of the head and neck were performed without normal controls, or used different type of samples as controls, *e.g*. tissue samples *versus* swabs 27 (Table 1). The lack of appropriately matched controls can lead to erroneous interpretations of the results. Namely, the detection of HPV DNA in tumours may be regarded as an aetiopathogenetically important infection, while it could in fact merely represent an incidental tissue colonization.

After our manuscript had been submitted for publication, an interesting paper was published by Patel *et al*. 45, using an additional methodological approach, further supporting the hypothesis that VC of the head and neck is not an α-PV induced tumour. Namely, no evidence of transcriptionally active high-risk α-PV was found in 18 cases of pure VC by RT-PCR for HPV E6/E7 mRNA. Other HPV genera were not examined in this study.

In conclusion, to the best of our knowledge, this is the largest study in which a broad spectrum of HPV types was comparatively analysed in VC of the head and neck and location-matched normal tissue controls, using highly sensitive and specific molecular assays. Our results suggest that α-PV, γ-PV and μ-PV are not associated with the aetiopathogenesis of VC. Even though the presence of β-PV DNA in a subset of VC and normal tissue samples could merely reflect an incidental tissue colonization, the potential biological significance of β-PV in VC needs further investigation. The list of HPV-induced SCC variants in the head and neck is increasing 3, but it appears that VC, despite its name, is not one of them.
